# Effect of Dietary Fatty Acids on Human Lipoprotein Metabolism: A Comprehensive Update

**DOI:** 10.3390/nu7064416

**Published:** 2015-06-02

**Authors:** Esther M.M. Ooi, Gerald F. Watts, Theodore W.K. Ng, P. Hugh R. Barrett

**Affiliations:** 1 Metabolic Research Centre, School of Medicine and Pharmacology, University of Western Australia, Perth, 6000, Western Australia, Australia; E-Mails: esther.ooi@uwa.edu.au (E.M.M.O.); gerald.watts@uwa.edu.au (G.F.W.); theodorewai.ng@uwa.edu.au (T.W.K.N.); 2 Lipid Disorders Clinic, Cardiometabolic Service, Department of Internal Medicine, Royal Perth Hospital, 6000, Western Australia, Australia; 3 Faculty of Engineering, Computing and Mathematics, University of Western Australia, Perth, 6000, Western Australia, Australia

**Keywords:** lipoprotein metabolism, dietary fatty acids, cardiovascular disease, dyslipidemia

## Abstract

Dyslipidemia is a major risk factor for cardiovascular disease (CVD). Dietary fatty-acid composition regulates lipids and lipoprotein metabolism and may confer CVD benefit. This review updates understanding of the effect of dietary fatty-acids on human lipoprotein metabolism. In elderly participants with hyperlipidemia, high *n*-3 polyunsaturated fatty-acids (PUFA) consumption diminished hepatic triglyceride-rich lipoprotein (TRL) secretion and enhanced TRL to low-density lipoprotein (LDL) conversion. *n*-3 PUFA also decreased TRL-apoB-48 concentration by decreasing TRL-apoB-48 secretion. High *n*-6 PUFA intake decreased very low-density lipoprotein (VLDL) cholesterol and triglyceride concentrations by up-regulating VLDL lipolysis and uptake. In a study of healthy subjects, the intake of saturated fatty-acids with increased palmitic acid at the sn-2 position was associated with decreased postprandial lipemia. Low medium-chain triglyceride may not appreciably alter TRL metabolism. Replacing carbohydrate with monounsaturated fatty-acids increased TRL catabolism. *Trans-*fatty-acid decreased LDL and enhanced high-density lipoprotein catabolism. Interactions between *APOE* genotype and *n*-3 PUFA in regulating lipid responses were also described. The major advances in understanding the effect of dietary fatty-acids on lipoprotein metabolism has centered on *n*-3 PUFA. This knowledge emphasizes the importance of regulating lipoprotein metabolism as a mode to improve plasma lipids and potentially CVD risk. Additional studies are required to better characterize the cardiometabolic effects of other dietary fatty-acids.

## 1. Introduction

Dyslipidemia is an independent and powerful risk factor for cardiovascular disease (CVD). Nutritional and lifestyle modifications are the frontline for the treatment of dyslipidemia to minimize and lower CVD risk. Current dietary recommendations focus on fatty acids and include reductions in saturated and *trans*-fatty acids, and an emphasis on consumption of mono- and poly-unsaturated fatty acids [1]. A better understanding of the mechanisms of action of these fatty acids is, therefore, a priority in contemporary cardiometabolic research. This review updates understanding of the mechanisms of action of individual dietary fatty acids on lipoprotein metabolism in hyperlipidemic humans, with a focus on studies that have employed stable isotope tracer methodologies and compartmental modeling (Table 1).

## 2. Polyunsaturated Fatty Acids

Recent advances in understanding the impact and use of dietary fatty acids in cardiometabolic medicine have focused on polyunsaturated fatty acids (PUFA), and reviewed below.

### 2.1. Omega-3 Polyunsaturated Fatty Acids

Fish oils are a rich source of the long-chain *n*-3 PUFA, eicosapentaenoic (EPA) and docosahexaenoic (DHA) acids. Compelling evidence suggest that *n*-3 PUFA reduces CVD risk and this is partly mediated by its potent triglyceride-lowering effects [2,3,4]. *n*-3 PUFA has been shown to reduce triglyceride synthesis via inhibition of diacylglycerol acyltransferase, fatty acid synthase and acetyl CoA carboxylase enzymes [4]. *n*-3 PUFA also enhance fatty acid β-oxidation via a peroxisome proliferator-activated receptor (PPAR)-α mediated pathway resulting in decreased substrate availability for triglyceride formation [4]. In addition, *n*-3 PUFA suppress transcription of sterol regulatory element-binding protein-1c gene and hence, inhibit *de novo* lipogenesis [4]. *n*-3 PUFA may also stimulate the post-endoplasmic reticulum presecretory proteolysis pathway, thereby increasing the degradation of newly synthesized apolipoprotein (apo) B [5]. These molecular mechanisms are consistent with *in vivo* studies. We observed that a high-fish diet (1.23 g/day EPA and DHA) decreased non-fasting plasma triglycerides, with concomitant reductions in triglyceride-rich lipoprotein (TRL) apoB-100 concentration and production rate, compared with a low-fish diet in elderly men and women with moderate hyperlipidemia [6] (Table 1). We also noted that a high-fish diet decreased TRL apoB-100 direct catabolism, and by implication, the conversion of TRL to low-density lipoprotein (LDL) was increased [6]. In addition, a high-fish diet decreased intestinally derived TRL apoB-48 concentration by decreasing TRL apoB-48 secretion. This finding is consistent with observations from *in vitro* and animal studies showing that *n*-3 PUFA suppressed TRL apoB-48 synthesis by either decreasing apoB-48 mRNA expression and/or increasing post-translational degradation of newly synthesized apoB-48 [7]. TRL-apoB-48 fractional catabolism was significantly decreased with the high-fish diet [6].The exact mechanism for the reduction in TRL apoB-48 catabolism is, however, unclear. It is possible that these particles were less triglyceride-enriched and were, therefore, poorer substrates for lipoprotein lipase. A more recent study from our group corroborated the effect of *n*-3 PUFA on TRL apoB-48 metabolism [8] (Table 1). Using a new compartmental model, we investigated the combined effects of weight loss and *n*-3 PUFA supplement compared with weight loss alone on TRL apoB-48 metabolism in a postprandial, non-steady state setting. The study was a 12-week, randomized intervention trial of a hypocaloric diet (energy deficit of ≥1900 kJ) and *n*-3 PUFA supplement (4 g/day Omacor; 46% EPA and 38% DHA) compared with a hypocaloric diet alone on TRL apoB-48 metabolism in obese men and women following ingestion of an oral fat load (a total of 4800 kJ, 130 g fat, 17 g protein, and 21 g carbohydrate) [8]. Our study showed that weight loss and *n*-3 PUFA supplement decreased fasting and total postprandial TRL apoB-48 area under the curve (AUC) compared with weight loss alone. These changes were chiefly due to a significant reduction in basal TRL apoB-48 secretion rate, without a significant effect during the postprandial period [8]. The exact reason for the lack of an effect on TRL apoB-48 during the postprandial period is unclear, but may relate to the efficient transport of lipid from the intestine into the portal system. 

While there is compelling evidence for CVD benefits from *n*-3 PUFAs, the contribution of the individual components, EPA and DHA, are not well understood. Both EPA and DHA effectively lower plasma triglyceride concentrations [9,10]. Only DHA increases LDL and HDL particle sizes, however [9,10], and the clinical significance of this remains unclear. EPA and DHA may also have different anti-hypertensive, anti-inflammatory and anti-coagulative properties; DHA being more effective compared with EPA [9,10]. This may, in part, explain the differential associations of EPA and DHA with risk of fatal and non-fatal cardiovascular endpoints in observational studies [9,10]. To date, no studies have investigated the independent impact of EPA and DHA on lipoprotein metabolism; such studies are warranted to clarify the lipid-modifying effects and therapeutic potential of consuming either EPA or DHA rich oils. 

Recent clinical outcome trials with *n*-3 PUFA have failed to show a significant CVD benefit [11,12,13,14], however. It is noteworthy that *n*-3 PUFA were taken against a background of optimal medical therapy for secondary prevention, for relatively short duration, and in patients that were at high-risk of CVD events. Whether a higher dose and/or specific formulation of *n*-3 PUFA may improve clinical outcomes remains to be established [2]. This will be addressed in an on-going trial (REDUCE-IT) to evaluate the effect of high dose of pure EPA (4 g/day) in preventing CVD events in high-risk patients with hypertriglyceridemia (NCT01492361). 

### 2.2. Omega-6 Polyunsaturated Fatty Acids

*n*-6 PUFA is the predominant dietary PUFA in Western populations. Therefore, understanding the impact of n-6 PUFA on lipid metabolism is clinically and scientifically relevant. A recent study by van Schalkwijk *et al.* examined the effect of 60 g/day of dietary *n*-6 PUFA (71% linoleic acid) compared with medium-chain fatty acid (MCFA; 69% C8:0 and C10:0) supplementation on fasting lipoprotein profile and metabolism in 12 overweight-obese men using a randomized, double-blind crossover study design [15] (Table 1). Three weeks supplementation with *n*-6 PUFA lowered fasting total, very low-density lipoprotein (VLDL) and LDL cholesterol, and total plasma, VLDL and LDL triglyceride concentrations compared with MCFA. Using a combination of the Particle Profiler computational model and compartmental modeling, the authors reported that the uptake to production ratio, but not lipolysis to production ratio, in VLDL was significantly higher with *n*-6 PUFA compared with MCFA supplementation [15]. When the information from the two ratios was combined, the authors reported that higher VLDL lipolysis and uptake explained the lower VLDL cholesterol and triglyceride concentrations with *n*-6 PUFA. The authors attributed this to an up-regulation of lipoprotein lipase (LPL) activity and hepatic uptake of VLDL, consequent on PPAR-activation by *n*-6 PUFA [15]. The study was not a controlled feeding study and used a non-standardized dietary protocol that may underestimate or bias the true effects of these fatty acids, however. Furthermore, there is limited insight into intermediate-density lipoprotein (IDL) and LDL metabolism owing to the short duration of the tracer infusion. The exact significance of a high *n*-6 PUFA diet on CVD outcomes also merits investigation.

## 3. Saturated Fatty Acids

A small number of studies have examined the impact of dietary saturated fatty acid (SFA) intake on lipoprotein metabolism in humans. Early radioisotope studies demonstrated that decreasing SFA, together with increasing *n*-6 PUFA, lowered LDL cholesterol by increasing LDL apoB-100 fractional catabolism [16,17] (Table 1). 

More recently, it was proposed that the physical characteristics of the triacylglycerol structure of SFA may impact on lipoprotein metabolism and consequently, lipemia. In a randomized crossover study, Sanders *et al. *examined whether meals high in palmitic acid, a major SFA in human diets, which is also synthesized *de novo*, altered postprandial lipemia [18]. The authors reported a positive trend between palmitic acid at the sn-2 position and the incremental AUC of plasma triglyceride. Their data suggest that as the proportion of palmitic acid at the sn-2 position increases, postprandial lipemia decreases. The postprandial plasma triglyceride concentration curve was also shifted to the right following the meal with the highest proportion of palmitic acid at the sn-2 position, but was not different by 6–8 h post-meal. The authors attributed this to delayed absorption of dietary triglycerides with increasing proportion of palmitic acid at the sn-2 position [18]. Kinetic studies are, nonetheless, required to establish whether the intake of SFA with altered triacylglycerol structures alters postprandial triglyceride absorption. 

The length of the SFA may also have differential effects on lipoprotein metabolism. Saturated fats may be sub-classified into short-chained (<6 carbon atoms), medium-chained (8–10 carbon atoms) and long-chained fatty acids (>12 carbon atoms). In a randomized crossover study, Tremblay *et al.* examined the effect of 4-week supplementation of 20 g of medium-chained fatty acid, also known as medium-chained triglyceride (MCT), compared with 20 g corn oil, on TRL apoB-100 and apoB-48 metabolism in obese, insulin resistant men [19] (Table 1). The authors reported no impact of MCT on TRL metabolism or genes associated with TRL metabolism [19]. This study, however, was relatively short-term, tested only one dose of MCT and focused only on insulin resistant men. LDL metabolism was also not investigated [19]. Long-term studies using different MCT doses are, therefore, merited. Outcome studies are also required before dietary recommendations can be made. 

## 4. Monounsaturated Fatty Acids

Few studies have examined the effect of dietary monounsaturated fatty acid (MUFA) on lipoprotein metabolism in humans. Zheng *et al.* reported that replacing 17% of total energy intake from complex carbohydrate with MUFA selectively stimulated the secretion of VLDL and IDL particles containing apoE and apoC-III, while suppressing the secretion of particles that did not [20] (Table 1). Consequently, the concentration of apoB-containing lipoproteins with apoE and apoC-III was higher with the MUFA diet compared with the carbohydrate diet [20]. The MUFA diet was also associated with increased VLDL and IDL apoB catabolism, although LDL apoB metabolism was not altered [20]. This study lends support to the potential benefits of increased MUFA and reduced complex carbohydrate in modulating lipoprotein metabolism. The study, however, was non-randomized and short-term, with an intervention period of only three weeks. Notably, this study compared two relatively healthy diets, both low in SFA, high in fiber and utilized primarily low glycemic foods. 

Recent studies using a lipidomics approach further demonstrate the potential impact of MUFA on lipoprotein metabolism and composition. Kien *et al.* reported that replacing palmitic acid with oleic acid lowered total and LDL cholesterol concentrations, and the LDL:HDL ratio [21] in men and women. In addition, replacing palmitic acid with oleic acid lowered rates of fatty acid oxidation, as measured using the respiratory exchange ratio (RER) in the fasting, but not fed, state. Whether this was associated with changes in the molecular regulation of fatty acid oxidation is, however, unclear as the relevant gene expression levels were only measured in the fed state [21]. Furthermore, it is unclear whether RER was measured under steady-state conditions given the short-term nature of the intervention and washout periods. Long-term studies with larger sample sizes are, therefore, warranted. 

### Trans-Fatty Acids

To date, only one study has examined the impact of *trans-*fatty acid (TFA) on lipoprotein metabolism. Five weeks consumption of TFA derived partially hydrogenated fats was associated higher total and LDL cholesterol, and lower HDL cholesterol concentrations in post-menopausal women [22]. These changes were attributed to impaired LDL apoB-100 and enhanced HDL apoA-I fractional catabolism (Table 1). Consumption of TFA did not alter TRL apoB-100 or apoB-48 metabolism [22]. Long- term studies, as well as studies in men and populations at higher cardiometabolic risk are warranted. Kinetics studies to better understand the impact of ruminant TFA, which are derived from natural sources such as meat and dairy products, and constitute a large proportion of TFA in the diet, are also awaited with interest [23,24]. 

**Table 1:** Summary of the current findings on the effect of dietary fatty acids on indices of lipoprotein metabolism in human.

Fatty Acids	Chemical Structure of Typical Fatty Acids	TRL apoB-48			VLDL apoB-100			IDL apoB-100			LDL apoB-100			HDL apoA-I			Ref No.
	(corresponding name)	Pool size	PR	FCR	Pool size	PR	FCR	Pool size	PR	FCR	Pool size	PR	FCR	Pool size	PR	FCR	
SFA	CH_3_(CH_2_)_16_COOH (stearic acid)										↓		↑				[16,17]
MCT	CH_3_(CH_2_)_10_COOH (lauric acid)	**≈**	**≈**	**≈**	**≈**	**≈**	**≈**										[19]
MUFA	CH_3_(CH_2_)_7_**CH=CH**(CH_2_)_7_COOH (oleic acid)				↓	↓	↑			↑	**≈**	**≈**	**≈**	**≈**	**≈**	**≈**	[20]
TFA	CH_3_(CH_2_)_7_**CH=CH**(CH_2_)_7_COOH (elaidic acid)	**≈**	**≈**	**≈**	**≈**	**≈**	**≈**				↑	≈	↓	↓	**≈**	↑	[22]
*n*-3 PUFA	CH_3_CH_2_**CH=CH**CH_2_**CH=CH**CH_2_**CH=CH**CH_2_**CH=CH**CH_2_**CH=CH**(CH_2_)_3_COOH (eicosapentaenoic acid)	↓	↓	↓	↓	↓	↑				↓	↑	↑	↓	↓	**≈**	[6,8]
*n*-6 PUFA	CH_3_CH_2_**CH=CH**CH_2_**CH=CH**CH_2_**CH=CH**CH_2_**CH=CH**CH_2_**CH=CH**CH_2_**CH=CH**(CH_2_)_2_COOH (docosahexaenoic acid)				↓	≈	↑										[15]

## 5. Dietary Fatty Acids and *APOE* Genotype Interaction

*APOE* is one of the more extensively investigated genes in CVD and *APOE* genotypes, E2, E3 and E4 are viewed as key genetic determinants of inter-individual variations in postprandial lipemia. In keeping with this concept, Liang *et al.* explored the interactions between *APOE* genotypes and plasma phospholipid EPA and DHA on plasma lipid and lipoprotein profiles in a cross-sectional analysis of 2340 Multi-Ethnic Study of Atherosclerosis (MESA) participants [25]. The authors reported significant *APOE* and EPA and DHA interactions with small-LDL concentration. Specifically, significant and positive associations were observed between EPA and DHA with small-LDL concentration in participants with E4, but not with E2 and E3. Therefore, the authors proposed that the interaction with LDL chiefly reflects a synergistic effect of the *APOE* E4 genotype and *n*-3 PUFA on LDL metabolism [25]. There is evidence that EPA and DHA may increase the conversion of VLDL to LDL particles. Furthermore, there is selective affinity of the apoE4 isoform for VLDL and hence, targeting the VLDL particle for preferential removal by the liver [26]. On balance, this may result in increased competition with LDL for hepatic receptors and consequently, delayed clearance and enhanced remodeling of LDL particles. The authors also reported significant *APOE* and phospholipid EPA interactions with HDL cholesterol and the concentrations of large and total HDL particles [25]. Specifically, there was a positive association with phospholipid EPA among the E2 participants and a negative association among the E4 participants. The authors, as before, proposed a synergistic effect between *APOE* E2 genotype and EPA on HDL metabolism [25]. The underlying mechanism, however, is not clear. Small increases in HDL cholesterol have been reported with *n*-3 PUFA supplementation, but whether this is secondary to plasma triglyceride-lowering is not known [27]. In addition, while apoE has been shown to facilitate the expansion of the cholesterol ester core of HDL in conjunction with the action of lecithin cholesteryl acyltransferase [28], no studies have demonstrated an apoE2 isoform dominant effect. Stable isotope tracer studies may better elucidate the mechanism the underscore the interactions between *APOE* genotype and lipid responses [29]. Further studies are also required to better understand the impact of *APOE* genotype and/or other genotypes on lipoprotein metabolism with dietary interventions.

## 6. Conclusions

The major advances in understanding the mechanisms of action of dietary fatty acids on lipoprotein metabolism have focused on *n*-3 PUFA. This knowledge provides insights into the importance of regulating lipoprotein metabolism as a means to improve the plasma lipid profile and lower CVD risk. Further studies are required to better understand the cardiometabolic effects of other dietary fatty acids. As part of this, consideration should be given to study design, dose and duration of intervention, and choice of food or nutrients substituted and those displaced, in order to optimally test a proposed hypothesis. Importantly, a holistic multi-nutrient approach, in particular healthy dietary patterns such as the Mediterranean diet, in place of individual nutrient substitution should also be applied and studied. This approach integrates the complexity of diet and potential interactions between nutrients and bioactive components and may further inform guidelines for CVD health and risk reduction. 
